# miRNAs: Important Targets for Oral Cancer Pain Research

**DOI:** 10.1155/2017/4043516

**Published:** 2017-10-30

**Authors:** Cláudia Maria Pereira, Dayany Sehnem, Estevão Oliveira da Fonseca, Heráclito Fernando Gurgel Barboza, Antônio Carlos Pires de Carvalho, Alexandre F. M. DaSilva, Vivaldo Moura-Neto, Marcos F. DosSantos

**Affiliations:** ^1^Programa de Pós-Graduação em Biomedicina Translacional, Universidade do Grande Rio (Unigranrio), Duque de Caxias, RJ, Brazil; ^2^Programa de Pós-Graduação em Odontologia Clínica e Experimental, Universidade do Grande Rio (Unigranrio), Duque de Caxias, RJ, Brazil; ^3^Universidade Federal do Rio de Janeiro (UFRJ), Campus Macaé, Macaé, RJ, Brazil; ^4^Sociedade Brasileira para o Estudo da Dor (SBED), São Paulo, SP, Brazil; ^5^Programa de Pós-Graduação em Radiologia, Universidade Federal do Rio de Janeiro (UFRJ), Rio de Janeiro, RJ, Brazil; ^6^Headache & Orofacial Pain Effort (HOPE), Department of Biologic and Materials Sciences & Michigan Center for Oral Health Research (MCOHR), School of Dentistry, University of Michigan, Ann Arbor, MI, USA; ^7^Instituto de Ciências Biomédicas (ICB), Universidade Federal do Rio de Janeiro (UFRJ), Rio de Janeiro, RJ, Brazil; ^8^Instituto Estadual do Cérebro Paulo Niemeyer (IECPN), Rio de Janeiro, RJ, Brazil

## Abstract

Pain is a symptom shared by an incredible number of diseases. It is also one of the primary conditions that prompt individuals to seek medical treatment. Head and neck squamous cell carcinoma (HNSCC) corresponds to a heterogeneous disease that may arise from many distinct structures of a large, highly complex, and intricate region. HNSCC affects a great number of patients worldwide and is directly associated with chronic pain, which is especially prominent during the advanced stages of oral squamous cell carcinoma (OSCC), an anatomical and clinical subtype that corresponds to the great majority oral cancers. Although the cellular and molecular bases of oral cancer pain have not been fully established yet, the results of recent studies suggest that different epigenetic mechanisms may contribute to this process. For instance, there is strong scientific evidence that microRNAs (miRNAs), small RNA molecules that do not encode proteins, might act by regulating the mechanisms underlying cancer-related pain. Among the miRNAs that could possibly interfere in pain-signaling pathways, miR-125b, miR-181, and miR-339 emerge as some of the most promising candidates. In fact, such molecules apparently contribute to inflammatory pain. Moreover, these molecules possibly influence the activity of endogenous pain control systems (e.g., opioidergic and serotonergic systems), which could ultimately result in peripheral and central sensitization, central nervous system (CNS) phenomena innately associated with chronic pain. This review paper focuses on the current scientific knowledge regarding the involvement of miRNAs in cancer pain, with special attention dedicated to OSCC-related pain.

## 1. Background

Head and neck squamous cell carcinoma (HNSCC), a prevalent and highly invasive cancer, is produced by unbalanced genetic and epigenetic events that take place in response to carcinogens. HNSCC affects a group of heterogeneous and intermingled structures, located in an incredibly complex region. HNSCC includes cancer in the oral cavity, larynx, and pharynx [1]. The main risk factors for HNSCC comprise behavior factors such as exposure to tobacco and alcohol abuse together with oncogenic viruses infections, especially human papilloma virus (HPV) [2]. Strikingly, less than 50% of the patients that develop oral or pharyngeal cancer survive for more than five years, according to a late-stage diagnosis [3]. Considering the several subsets of malignant tumors in the head and neck, squamous cell carcinomas (SCCs) originated from the oral mucosa (OSCC) and oropharynx (OPSCC), ranked as the 14th most prevalent type of cancer among women and the eighth among men in the US [4]. OSCC accounts for approximately 80–90% of all malignancies of the oral cavity and preferentially affects the following anatomic sites: tongue, retromolar region, floor of the mouth, hard palate, and jugal mucosa [5]. Not surprisingly, roughly 75% of all OSCC cases are credited to tobacco exposure or alcohol abuse [6]. More recently, HPV infection has also been linked to a subset of OSCC [7] that exhibits a higher incidence in younger patients [4] and that is often associated with improved prognoses [8].

**Figure 1:**
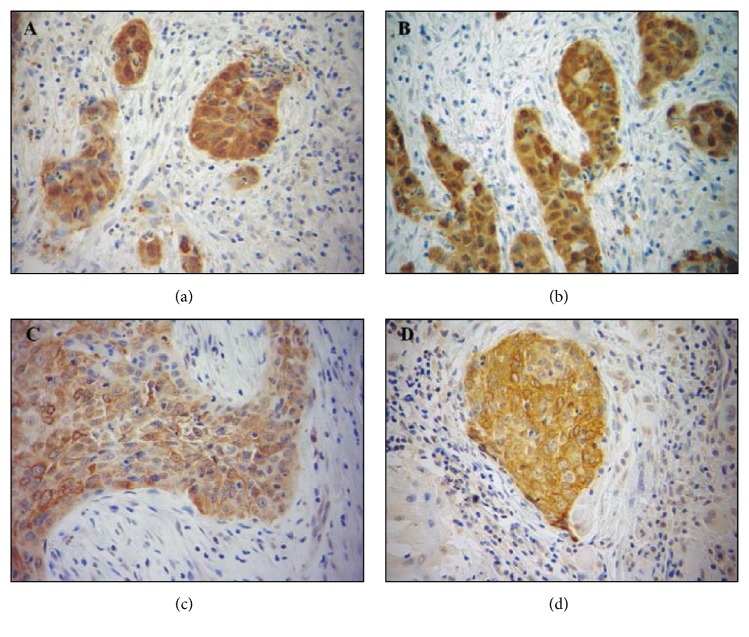
Immunohistochemical staining for MAGE A1 in oral squamous cell carcinoma (OSCC) samples. Cytoplasmic staining pattern in OSCC14 (a and b) and OSCC18 (c and d).

Notwithstanding substantial progress in the OSCC treatment has been witnessed in recent years, the effectiveness of the available therapies and recurrence are still relevant issues. Hence, there is a clear need for the discovery of new targets as well as signaling molecules in order to tailor more appropriate therapeutic strategies. Remarkably, it has been demonstrated that the overexpression of epidermal growth factor receptor (EGFR), a member of the ErbB receptor family, is associated with lower local tumor control after radiotherapy and a shortened overall survival in HNSCC [9, 10]. Additionally, other markers have been investigated as possible immunotherapy targets for OSCC treatment [11]. For instance, the expression of cancer testis antigen (CTA) genes like MAGE A1 has been described in HNSCC [12] and in OSCC cells [11] but not in the normal oral mucosa cells (Figure 1).

HNSCC treatment is also challenging due to the considerable discrepancies in the therapeutic response among individuals. Therefore, the discovery and validation of novel biomarkers such as microRNAs (miRNAs) will contribute to more accurate treatment monitoring as well as the development of customized therapies, both with a direct impact on patients' quality of life [13]. In fact, changes in the circulating miRNAs have been found during the course of HNSCC radiochemotherapy, suggesting that some of those molecules (e.g., miR-425-5p and miR-93-5p) can be used as prognostic indicators, when evaluating the effects of cancer therapeutics [14]. Furthermore, according to a recent study, some polymorphisms in pre-miRNAs might influence the prognosis of squamous cell carcinomas of the nonoropharynx, particularly in patients undergoing chemoradiation and in smokers [15].

The presence of spontaneous pain prior to cancer therapy is also an important feature that must be considered when evaluating the prognosis of OSCC [16]. Nonetheless, despite the recent boom of research studies dedicated to find reliable biomarkers that might impact the response to the therapies and, therefore, the prognosis of OSCC [17–21], the number of studies that focus on the characterization of the molecular events related to oral cancer pain is still very limited. This fact might be explained by the relative low incidence of pain during the early stages of OSCC though its occurrence significantly increases over the course of the disease, becoming more pronounced at its later phases [22, 23]. According to some authors, the presence of pain in the early stages of OSCC depends on the anatomical site of the lesion, the activity of proinflammatory cytokines secreted by the invasive tumor cells, the presence of perineural/muscular invasion, and, finally, the existence of lymphoplasmacytic infiltration [16, 22, 24].

## 2. Pain in Cancer Patients

Cancer-related pain represents a daunting challenge for both clinicians and pain researchers all over the globe. In fact, pain is among the concerning cancer symptoms, especially when considering the extremely negative impact that it exerts not only in the physical but also in the emotional state and cognitive functioning of the patients afflicted by such disease. Different subtypes of HNSCC have been connected to pain, which in many occasions can be excruciating and disabling, thus producing an tremendous deleterious influence over the quality of life and the functional activities of the individuals affected [25]. Overall, it has been estimated that around one-third of the patients that receive treatment for cancer and two-thirds of those subjects who are in the advanced stages of the disease experience pain. Likewise, both the inflammatory and the neuropathic pain components may be present, contributing to its challenging clinical management [26].

A multiplicity of tumors (e.g., carcinomas and sarcomas) is associated with osteogenic metastasis and ends up producing spontaneous bone pain [11], a symptom that should receive considerable attention from clinicians. In fact, cancer patients that report pain throughout the clinical evaluation exhibit a considerable reduction of the pain threshold, explaining the presence of several pain phenomena routinely witnessed in those patients. For example, in some cases, even innocuous stimuli are capable of eliciting pain, a phenomenon defined as allodynia. In addition, an amplification of the painful response to harmful stimuli in a damaged tissue (primary hyperalgesia) or in the surrounding areas (secondary hyperalgesia) might be reported as well and strongly suggest that peripheral as well as central sensitization might take part in this process. These phenomena are often accompanied by a considerable increase in the levels of anxiety and depression [26, 27].

As previously described, although OSCC is usually asymptomatic during its initial stages, as in other types of cancer, pain might emerge during the subsequent phases. When present, functional pain remarkably surpasses spontaneous pain in OSCC patients [28]. Moreover, the presence of spontaneous pain before cancer treatment might predict an unfavorable prognosis of OSCC [16]. As a matter of fact, as previously reported, higher pain levels, which can be easily assessed at the clinical setting by the application of a visual analogue scale (VAS), predict the existence of perineural invasion in OSCC. Hence, a simple baseline pain evaluation allows clinicians to obtain a more accurate prognosis and to achieve an enhanced treatment decision in cancer patients [29].

Notwithstanding numerous past studies have confirmed the incidence of referred pain in cancer patients [9, 14], its mechanisms remain largely unveiled. The elucidation of the cellular, biochemical, and molecular aspects of cancer pain is crucial to the identification of the events related to this phenomenon. Thus, future therapeutic strategies for OSCC-related pain should rely not only on the signs and symptoms of the individuals affected but also on the pathophysiological mechanisms and dysfunctional signaling pathways implicated in the whole process. In this context, recent studies suggest that epigenetics (e.g., DNA methylation and miRNA) is a fundamental step in the deleterious transition from acute to chronic pain [30].

## 3. How Are miRNAs Involved in Pain Mechanisms?

miRNAs are a class of small RNAs (18–22 nucleotides) that do not encode proteins [31]. Nonetheless, those molecules do mediate gene expression through mechanisms of degradation or inhibition of the messenger RNA (mRNA) [32–34]. miRNAs can be actively exported from the intracellular to the extracellular space and this mechanism seems to be part of a complex response system that involves the participation of miRNAs in the cell-to-cell communication [35]. Slight fluctuations in the expression of signaling molecules, ion channels, and structural proteins, which in turn are directly linked to miRNAs activity, are inherently associated with the development and maintained of chronic pain [36].

A possible contribution of miRNAs to pain mechanisms has arisen few years ago, when a hypoexpression of miRNAs was demonstrated in the trigeminal ganglion neurons of an experimental model of inflammatory myogenic pain, clearly demonstrating that miRNAs can act at the peripheral nervous system and that changes in the expression of such key molecules can be related to the development of both allodynia and hyperalgesia [37].

In order to determine a signature of miRNAs associated with cancer pain, a previous study performed a wide genome-wide screening of miRNAs in sensory neurons [26]. Among the set of miRNAs that exhibited altered expression, some miRNAs were validated as important modulators of tumor-associated hypersensitivity. Those findings support the investigation of miRNAs' contributions to chronic pain syndromes, including pain induced by cancer.

## 4. Evidence of miRNAs Participation in Cancer-Related Pain?

Despite the still scarce number of clinical studies, the scientific literature available suggests that epigenetic changes in the brain, as well as in the spinal cord, occur in different chronic pain conditions [27, 38–40]. Supporting this hypothesis, changes in the circulating miRNAs expressions have been previously demonstrated in different painful syndromes such as complex regional pain syndrome (CRPS) [41], fibromyalgia [42], irritable bowel syndrome [43], endometriosis [43], and osteoarthritis [44].

In irritable bowel syndrome (IBS), a disease with unclear mechanisms and difficult clinical management, the primary clinical visceral pain was correlated to a decreased expression of the colonic miR-199a/b in a previous study. Moreover, the upregulation of miR-199a reduced the IBS-related visceral pain [45]. In another study, an alternative pattern was demonstrated in migraine patients, wherein an acute upregulation of the miR-34a-5p and miR-382-5p expression was found during the migraine attacks [46]. Furthermore, miR-382-5p was overexpressed in migraine patients when compared to controls, not only during the headache attacks, but also during the pain-free periods. Interestingly, the same miR-382-5p is considered a brain-enriched miRNA, predominantly located in neurons and cerebrospinal fluid (CSF). Considering the possible involvement of a blood brain barrier (BBB) leakage in the migraine pathophysiology [47], it would be reasonable to consider that the increased concentrations of miR-382-5p observed in the serum could be derived from the CNS or the CSF [46]. Nevertheless, it is important to mention that though at lower concentrations, the miR-382-5p can be naturally detected in the serum [48].

Although the specific mechanisms whereby miRNAs contribute to nociception and pain are still not clear, there is mounting evidence that adaptive changes in the miRNAs expressions in response to tissue injury influence the activity of proinflammatory cytokines, which in turn mediate acute inflammatory pain [27, 49, 50]. Based on this concept, it is possible to hypothesize that a similar process underlies cancer pain.

In fact, it has been recognized that inflammatory mechanisms play a role in cancer establishment and progression. Those mechanisms potentially activate pain-signaling pathways. For example, a typical inflammatory response is characterized by an increased production of inflammatory mediators, including prostaglandin E2 (PGE2), interleukin 1 beta (IL-1*β*), and tumor necrosis factor-alpha (TNF-*α*) which also contribute to the development of hyperalgesia [51]. The same cytokines can affect the expression of miRNAs [52, 53] and might be modulated by miRNAs [54]. This hypothesis is sustained by the altered miRNAs regulation that occurs in metastatic bone areas, which was previously demonstrated in an experimental animal model of cancer pain. Moreover, the miRNAs targets found in the same study comprised pain-related genes [26].

All this growing evidence reinforces the promising use of miRNAs as potential targets in future pain therapies [55]. For instance, a previous work described that the increased inflammatory visceral pain in conjunction with the expression of the transient receptor vanilloid type 1 (TRPV1) was correlated to a decreased Gut miR-199a/b expression in IBS. Conversely, the upregulation of miR-199a reduced the IBS visceral pain through the inhibition of the TRPV1 signaling, indicating that miR-199a precursors might be clinically tested to the treatment of inflammatory visceral pain and in particular IBS [45].

Some promising miRNAs such as miR-125b-3p [56], miR-125b-5p [42, 57], miR-181 [58], miR-30d-5p, miR-379-5p [57], and miR-339 [41] have been correlated to pain. Among the predicted targets for these miRNAs are some of the chief molecules for chronic pain, including the calcitonin gene related peptide (CGRP) [59, 60], TNF-*α* [61], transforming growth factor beta receptor 1 (TGFBR1) [62, 63], toll-like receptor (TLR-4) [64], interleukin 1 alfa (Il-1*α*) [65], interleukin 10 receptor, alpha subunit (IL-10RA) [66], and interleukin 6 receptor (IL-6R) [67]. Using genome‐wide miRNA screening and molecular and in silico analyses, a previous study identified a subset of 57 miRNAs associated with metastatic bone-cancer pain in mice and validated miRNAs 1a‐3p, 34c‐5p 483‐3p as important modulators of tumor-associated hypersensitivity [26]. A more recent study that applied in-depth bioinformatics analysis identified the Cav 2.3 as a target for miR-34c‐5p [68]. Moreover, knockdown of Cav 2.3 in the dorsal root ganglion (DRG) of mice resulted in hypersensitivity, which clearly indicates an antinociceptive role of this calcium channel in sensory neurons.

Inflammation is also an important process related to the major cancer-related symptoms, including pain [69]. Not surprisingly, some miRNAs that are involved in the mechanisms of inflammation, including miR-181 family members as well as miR-125b, have targets that are also important to the nociceptive transmission. Indeed, a basic search using computer-based programs available for miRNA target prediction such as microRNA.org [70] and TargetScanHuman 7.1 [71] reveals that miR-181a-5p as well as mir-181b-5p might bind to the gamma-aminobutyric acid type A receptor, alpha 1 subunit (GABRA1). Supporting this information, an upregulation of miR-181a along with a downregulation of GABRA1 gene and protein expression in the dorsal horn of the spinal cord has been demonstrated in an experimental model of chronic inflammatory pelvic pain induced by neonatal cystitis [58]. Based on these results, it has been hypnotized that this downregulation of GABAA receptors induced by miR-181a could lead to a decreased inhibition in the spinal cord dorsal horn, contributing to a persistent visceral hypersensitivity [72].

The miR-181 family is composed by four members (miR-181a, miR-181b, miR-181c, and miR-181d). These miRNAs are considered extremely important in the regulation of inflammation under physiological and pathological conditions [73]. In a previous study, miR-181a was able to inhibit the activity of the proinflammatory cytokine interleukin 8 (IL-8) of inflamed dental pulp tissue [74]. Thus, it is possible to speculate that this miRNA is directly involved in the regulation of pain and inflammation in the orofacial tissues. On the other hand, miR-125b-3p has been implicated in neuropathic as well as inflammatory pain, raising the possibility of contribution for cancer-related pain [59]. Variations of miR-125b-3p expression have also been observed in the hippocampus of animals submitted to surgical chronic constriction of the sciatic nerve, a widely used experimental model of neuropathic pain [56]. Those results bring the intriguing question of whether a synaptic plasticity regulated by slight variations of miRNAs' expression in specific areas of the peripheral/central nervous system could also be essential for neuropathic pain development.

Likewise, miR-339 seems to be important for cancer-related pain. miR-339-3p is a natural target of mu-opioid receptors. Those receptors are part of the opioidergic system, an important endogenous modulatory system and target of the great majority of the opioid drugs available for pain control. Those include morphine and related agonists, which are frequently used for cancer pain management [75–78].

**Figure 2:**
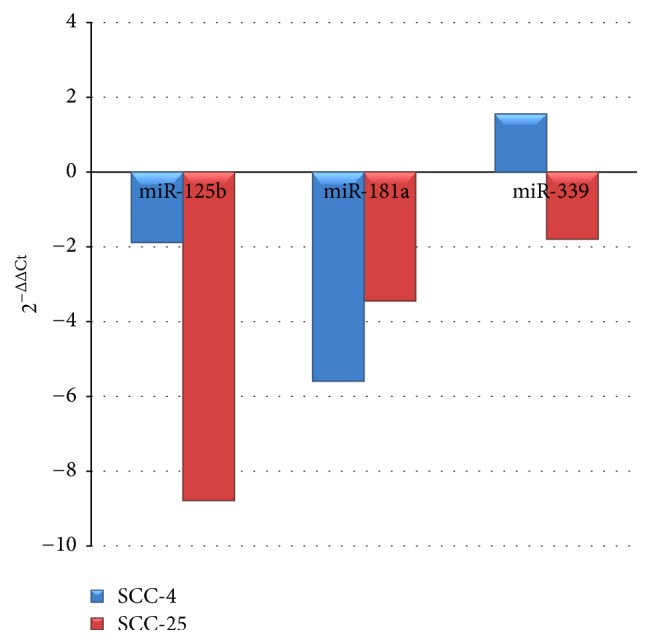
*Expression of miR-125, miR-181, and miR-339 in OSCC cell lines*. miRNAs expressions in SCC-4 (blue) and SCC-25 (red) compared to a immortalized keratinocyte cell line (HaCaT). These cell lines were obtained from the Rio de Janeiro Cell Bank (Banco de Células do Rio de Janeiro, BMCRJ) which originates from American Type Culture Collection (ATCC).

An explorative analysis performed in OSCC cell lines reveals a marked change in the expressions of miR-125, miR-181, and miR-339 in OSCC cell lines, when compared to normal keratinocytes (Figure 2, unpublished data). This preliminary finding corroborates the participation of such molecules on the OSCC pathophysiology, demonstrated in previous studies. For instance, it has been reported that the overexpression of miR-181a and miR-181b may increase lymph-node metastasis, vascular invasion by tumor, and poor prognosis in OSCC patients, suggesting that this miRNA could be a potential biomarker of this disease [79]. Besides, downregulation of miR-125b has been found in OSCC cell lines and tumors and the transfection of the cells with exogenous miR-125b decreased cell proliferation and affected the expression of cancer-related genes [80]. Nonetheless, despite the scientific evidence supporting the involvement of these miRNAs in both neuropathic and inflammatory cancer-related pain and their possible participation in the OSCC pathophysiology, it is still not possible to completely determine to which extent these findings are correlated to the clinical presentation and OSCC-induced pain. Therefore, further studies will be necessary to confirm this information and to expand the current knowledge regarding the miRNAs participation in chronic pain and particularly in cancer-related pain. In this context, miR-199, miR-34a-5p, and miR-382-5p are promising molecules that must be investigated in depth. In addition, other important topics in the field must be addressed in future works, such as the contribution of miRNAs to the development of mucositis, a common effect of cancer therapy, that is usually characterized by excruciating pain, which in turn is often reported as the most prominent and disturbing symptom during the treatment for head and neck cancer [81, 82].

## 5. Conclusions

miRNAs have been considered potential biomarkers and possible therapeutic targets in a wide spectrum of clinical disorders, including chronic pain syndromes, with especial interest to cancer-related pain. Although numerous studies have improved the current understanding regarding the clinical manifestations of cancer-related pain, there remains unknowns concerning its pathophysiology as well as the disrupted signaling pathways involved. Therefore, the clarification of the cellular, biochemical, and molecular aspects related to cancer-induced pain is a fundamental step to the identification of the complex cascade of events that lead to this phenomenon. In the future, this knowledge will potentially allow the development of novel therapeutic strategies based on the dysfunctional mechanisms of cancer pain and not merely on the signs and symptoms reported. In this context, upcoming studies devoted to scrutinize the epigenetics of OSCC and other types of cancer, correlating such results to the clinical findings, will be crucial to evolve the clinical management of oral cancer-related pain.

## Abbreviations

BBB: Blood brain barrier
CNS: Central nervous system
CGRP: Calcitonin gene related peptide
CRPS: Complex regional pain syndrome
CSF: Cerebrospinal fluid
CTA: Cancer testis antigen
GABRA1: Gamma-aminobutyric acid type A receptor, alpha 1 subunit
HNSCC: Head and neck squamous cell carcinoma
IBS: Irritable bowel syndrome
Il-1*α*: Interleukin 1 alfa
IL-1*β*: Interleukin 1 beta
IL-6R: Interleukin 6 receptor
IL-8: Interleukin 8
IL-10RA: Interleukin 10 receptor
mRNA: Messenger RNA
miRNAs: microRNAs
OSCC: Oral squamous cell carcinoma
PGE2: Prostaglandin E2
TGFBR1: Transforming growth factor beta receptor 1
TLR-4: Toll-like receptor
TNF-*α*: Tumor necrosis factor-alpha
TRPV1: Transient receptor vanilloid type 1
VAS: Visual analogue scale.
SCC: Squamous cell carcinoma
OPSCC: Oropharyngeal squamous cell carcinoma.
